# Schizophrenia and the dynamic genome

**DOI:** 10.1186/s13073-017-0416-2

**Published:** 2017-03-02

**Authors:** Patrick F. Sullivan

**Affiliations:** 1 Department of Medical Epidemiology and Biostatistics, Karolinska Institutet, SE-17177 Stockholm, Sweden; 2 Department of Genetics, University of North Carolina, 120 Mason Farm Road, 5000 D, Genetic Medicine Building, Chapel Hill, NC 27599-7264 USA; 3 Department of Psychiatry, University of North Carolina, 101 Manning Drive, Chapel Hill, NC 27599-7264 USA

## Editorial summary

Copy number variation (CNV) is a widely replicated risk factor for psychiatric disorders such as schizophrenia, although the mechanisms by which CNVs confer risk are currently unclear. Recent studies have provided robust evidence of CNVs associated with schizophrenia and have highlighted a potential role for schizophrenia risk-associated CNVs in impairing inhibitory learning; these studies highlight how insights can be gained into the associations of CNVs with schizophrenia.

## Copy number variation and the human genome

The human genome is not static, and changes of many types and at many scales occur in the creation of every generation. One of the more interesting types of genetic alteration is copy number variation (CNV). Germline CNVs occur with some regularity, and certain genomic regions are more prone to developing CNVs than others owing to an increased chance of errors occurring at certain locations during meiosis [1]. Most CNVs are small and have no impact, but larger CNVs can change the number of copies of one or more genes from the normal inherited complement.

The impact of a CNV depends on the gene, as some genes can be duplicated or deleted with no observable effect. Some CNVs can even provide benefit or protection against environmental exposures [2]. However, the function of some genes is exquisitely sensitive to dosage. A CNV that leads to an increase or a decrease from the normal inherited complement of a gene that is important to the development or function of the central nervous system can markedly increase the risk of an individual developing a psychiatric disorder such as schizophrenia or autism [3].

Two recent reports [4, 5] have markedly augmented our knowledge of schizophrenia and, as with all worthy papers, they answer some questions and raise intriguing new ideas.

## Copy number variation in schizophrenia

The first unequivocal genetic risk factor for schizophrenia was a CNV. In the 1990s, several groups described a strong association of a one-copy loss of a three million base pair region on chromosome 22 [6]. This deletion of 22q11 leads to a multisystem disorder known as velocardiofacial syndrome or 22q11 deletion syndrome, and individuals with this disorder have an increased risk of developing multiple conditions including schizophrenia. The basic association has replicated well [4], and the deleted region contains more than 50 genes, many of which are expressed in brain. This discovery has led to large-scale searches for CNVs associated with schizophrenia. Multiple studies have been undertaken, but a major issue is that the genomic signals being searched for can be subtle, variable, and difficult to replicate. Quality control for CNV studies is also notoriously complex, and it can be extremely challenging to identify robust findings.

The pinnacle of CNV searches for schizophrenia to date is a study from the CNV Working Group of the Psychiatric Genomics Consortium, which was led by Jonathan Sebat [4]. Although I am a co-author on this paper, this work is arguably the most careful and comprehensive study of CNVs that has so far been conducted in human genomics. The reason for this is that the authors took exceptional care in processing the data. Beginning with intensity files for 21,094 schizophrenia cases and 20,227 controls, they applied multiple CNV calling programs with careful quality control. This was a massive effort, and the study took a team of scientists more than 4 years to complete.

The authors reported that people with schizophrenia carry a mean of 11% more CNVs than do controls, and schizophrenia-associated CNVs particularly affected genes involved in synaptic function. They found strong evidence that eight CNVs were associated with schizophrenia and noted suggestive support for eight more. Most of these CNVs had already been reported, but the authors presented evidence that there are more CNVs to be discovered. The CNVs discovered to date are the ‘low-hanging fruit’ and have been relatively easy to detect owing to their large sizes, their relatively high prevalence in cases, and their scarcity in controls. Methods based on single nucleotide polymorphism arrays may be missing smaller or more complicated CNVs, the identification of which will require whole-genome sequencing. Larger studies, which are being planned by Sebat and colleagues, could also identify more CNVs.

## CNVs and the pathophysiology of schizophrenia

A crucial and as yet unanswered question is how each of these CNVs connects to the pathophysiology of schizophrenia. CNVs that affect the neurexin 1 gene (*NRXN1*) increase the risk of an individual developing schizophrenia [7], and neurexin 1 is critical to the formation and function of synapses [8]. Sekar et al. [9] studied a copy number polymorphism in the gene encoding complement component 4 (*C4*). They found that *C4* alleles tracked with risk of schizophrenia and showed that C4 mediated synapse elimination in a partially relevant mouse developmental model. The findings implicate complement activity in synaptic pruning (that is, the streamlining of synaptic connections) in schizophrenia.

CNVs that affect single genes such as *NRXN1* and *C4* are somewhat more tractable than are large CNVs, which may contain dozens of genes. The study by Clifton et al. [5] applied a clever alternative strategy to address this issue by exploring the molecular neurobiology of associative learning. Abnormalities in associative learning have been associated with schizophrenia, and the authors investigated whether the presence of schizophrenia-associated CNVs might be connected with specific phases of associative learning. Using a rat model, the authors looked for changes in gene expression during consolidation, retrieval, or extinction (that is, a decline in the conditioned response) of associative memories such as fear. After statistically assessing gene expression in the rats, they identified differentially expressed genes and their homologous human counterparts. The authors asked whether the genes connected with these memory-related processes were also found in CNVs that were more common in patients with schizophrenia than in healthy controls. The authors assumed that the rat and human genes had similar functions; although there certainly are differences between rats and humans that have arisen in the approximately 65 million years since the last common ancestor, many neurobiological processes are highly conserved. The authors’ analyses implicated genes involved in fear extinction, but not those involved in consolidation or retrieval. The results of this study suggest that CNVs impair inhibitory learning and may contribute to the development of some of the psychotic symptoms of schizophrenia.

This is a fascinating result as it suggests that rodent gene expression studies that use careful behavioral paradigms (which could never be carried out with humans) can propose directed hypotheses for future experiments. *It also suggests that fear may be a crucial part of schizophrenia*.

We certainly know that patients with psychosis are often intensely afraid owing to the content of their hallucinations or delusions (for example, a persecutory delusion that one is being incessantly and maliciously observed). Fear can also be a common factor in the experience of stressful life events. The authors raise the intriguing possibility that the powerful emotion of fear and the way in which fear is processed may be more fundamental to schizophrenia than we currently think. As with all good studies, addressing some hypotheses raised more new questions. The application of single-cell RNA sequencing has proven to be exceptionally informative in neuroscience, and it would be intriguing to see its application here. It might also be interesting to investigate whether persistent, unpredictable stress (which may induce fear) during pubescence leads to lasting changes in adulthood.

These two intriguing studies [4, 5] suggest a larger lesson: that we are entering a golden age of schizophrenia research. Many research groups are working on CNVs that predispose to schizophrenia with approaches ranging from functional genomics to molecular neuroscience to evaluating patient-derived neuronal models. CNVs are complex, but these recent studies have highlighted how insights can be gained into their associations with schizophrenia risk. One can hope that progressive increases in knowledge will become the norm for this debilitating psychiatric disorder.

## Abbreviations

CNV: Copy number variation
